# Expanded Human Blood-Derived γδT Cells Display Potent Antigen-Presentation Functions

**DOI:** 10.3389/fimmu.2014.00344

**Published:** 2014-07-23

**Authors:** Mohd Wajid A. Khan, Stuart M. Curbishley, Hung-Chang Chen, Andrew D. Thomas, Hanspeter Pircher, Domenico Mavilio, Neil M. Steven, Matthias Eberl, Bernhard Moser

**Affiliations:** ^1^Institute of Infection and Immunity, Cardiff University School of Medicine, Cardiff, UK; ^2^NIHR Biomedical Research Unit, Centre for Liver Research, University of Birmingham Medical School, Birmingham, UK; ^3^Department of Immunology, Institute of Medical Microbiology and Hygiene, University of Freiburg, Freiburg, Germany; ^4^Unit of Clinical and Experimental Immunology, Humanitas Clinical and Research Center, Rozzano, Milan, Italy; ^5^Department of Medical Biotechnologies and Translational Medicine, University of Milan, Milan, Italy; ^6^CR-UK Clinical Trials Unit, School of Cancer Sciences, University of Birmingham Medical School, Birmingham, UK

**Keywords:** γδT cells, antigen-presentation, vaccine, αβT cells

## Abstract

Cell-based immunotherapy strategies target tumors directly (via cytolytic effector cells) or aim at mobilizing endogenous anti-tumor immunity. The latter approach includes dendritic cells (DC) most frequently in the form of *in vitro* cultured peripheral blood monocytes-derived DC. Human blood γδT cells are selective for a single class of non-peptide agonists (“phosphoantigens”) and develop into potent antigen-presenting cells (APC), termed γδT-APC within 1–3 days of *in vitro* culture. Availability of large numbers of γδT-APC would be advantageous for use as a novel cellular vaccine. We here report optimal γδT cell expansion (>10^7^ cells/ml blood) when peripheral blood mononuclear cells (PBMC) from healthy individuals and melanoma patients were stimulated with zoledronate and then cultured for 14 days in the presence of IL-2 and IL-15, yielding γδT cell cultures of variable purity (77 ± 21 and 56 ± 26%, respectively). They resembled effector memory αβT (T_EM_) cells and retained full functionality as assessed by *in vitro* tumor cell killing as well as secretion of pro-inflammatory cytokines (IFNγ, TNFα) and cell proliferation in response to stimulation with phosphoantigens. Importantly, day 14 γδT cells expressed numerous APC-related cell surface markers and, in agreement, displayed potent *in vitro* APC functions. Day 14 γδT cells from PBMC of patients with cancer were equally effective as their counterparts derived from blood of healthy individuals and triggered potent CD8^+^ αβT cell responses following processing and cross-presentation of simple (influenza M1) and complex (tuberculin purified protein derivative) protein antigens. Of note, and in clear contrast to peripheral blood γδT cells, the ability of day 14 γδT cells to trigger antigen-specific αβT cell responses did not depend on re-stimulation. We conclude that day 14 γδT cell cultures provide a convenient source of autologous APC for use in immunotherapy of patients with various cancers.

## Introduction

Dendritic cells (DC) are master regulators of adaptive immunity and are long thought to be a prime target for the treatment of various diseases, including chronic infections, autoimmune diseases, and cancer (1, 2). In principle, DC-based immunotherapies involve two separate strategies: infusion of patients with *in vitro* generated, vaccine-loaded DC and injection of patients with biologicals targeting the patients’ own DC (3). The former approach has the advantage of modifying *in vitro* cultured DC prior to their use as cellular vaccine. However, DC do not grow during *in vitro* culture and are scarce in peripheral blood. Therefore, a common strategy involves the *in vitro* generation of DC by culturing blood-derived monocytes for 6 days in the presence of IL-4 and GM-CSF [monocyte-derived DC (moDC)] (4). Again, this method does not yield unlimited numbers of moDC as the majority of cells die during the *in vitro* differentiation process. A hallmark of DC is their exquisite functional diversity underscored by the numerous distinct DC subsets present in blood and peripheral tissue and their varied reactivity to maturation factors, including cytokines and microbial stimuli (5). These multiple factors may have limited the use of DC-based cellular vaccines in the clinic, explaining the paucity in approved cell products [except for Sipuleucel-T (6)], despite decades of fundamental and clinical research (3).

γδT-antigen-presenting cells (APC), activated γδT cells with antigen-presentation function, might be a valuable alternative to moDC for use as cellular vaccines in the treatment of patients with cancer (7). γδT-APC are generated during short-term activation of human peripheral blood γδT cells expressing Vγ9Vδ2-TCR. This particular γδT cell subset predominates in peripheral blood (1–5% of total T cells) and recognizes a class of non-peptide ligands, so-called “phosphoantigens.” The most potent phosphoantigen, (*E*)-4-hydroxy-3-methyl-but-2-enyl pyrophosphate (HMBPP), is of microbial origin and outperforms the structurally related phosphoantigen found also in higher organisms, isopentenyl pyrophosphate (IPP), by >10^3^-fold (8). Recent evidence underscores the importance of butyrophilin 3A1/CD277 in presenting HMBPP or IPP to Vγ9Vδ2-TCR^+^ γδT cells, and differences in binding kinetics may explain the striking difference in their potency to induce cell responses (9, 10). Activation of Vγ9Vδ2-TCR^+^ γδT cells by HMBPP/IPP or aminobisphosphonates (nBP) that increase intracellular IPP levels due to inhibition of IPP-metabolizing enzymes, leads to multiple cell responses, including cytokine and chemokine secretion as well as tumor cell lysis (11–13). Indeed, the broad range of tumor cells that are being killed during *in vitro* culture with Vγ9Vδ2-TCR^+^ γδT cells (abbreviated hereafter as γδT cells) provided the rationale for targeting these cells in current cancer immunotherapy trials (14, 15).

We here propose to explore the DC-like APC properties of γδT cells and to discuss the possibility of translating our findings into a novel type of cellular vaccine. The principles underlying the two γδT cell-based translational approaches, i.e., tumor cell-killing and αβT cell activation, differ fundamentally from each other. Most notably, tumor cell-killing requires that infused γδT cells reach the sites of tumors in order to kill tumor cells during cell-to-cell contact. By contrast, the APC properties of γδT cells target endogenous αβT cells and, in order to do so, tumor-antigen-presenting γδT cells need to interact with tumor-specific αβT cells within secondary lymphoid tissues (spleen, lymph nodes). We do not anticipate that the mobilization of tumor-specific αβT cells in spleen and lymph nodes is hindered by those γδT cells that home to the tumor tissue. In fact, it may well be that tumor cell-killing by itself leads to *in situ* prepared tumor-antigen-presenting γδT cells that may further enhance endogenous αβT cell responses.

What is the evidence for DC-like properties of activated γδT cells? Similar to tumor cell-killing, the APC functionality is the result of extensive *in vitro* studies facilitated by the fact that human peripheral blood γδT cells uniformly respond to HMBPP/IPP. Resting peripheral blood γδT cells express receptors for inflammatory chemokines and, similar to T_EM_ cells, are in pole position to be recruited to sites of inflammation (16–19). However, during short-term (1–2 days) activation with IPP, the inflammatory homing program in γδT cells is switched to a transient lymph node-homing program characterized by CCR7 expression, suggesting their contribution to lymph node activities (16). In addition to cytokine production, IPP stimulation results in surface expression of multiple receptors commonly associated with DC, including antigen-presentation molecules (MHC class II), co-stimulatory receptors (CD40, CD80, CD86), maturation markers (CD83), and adhesion receptors (CD11a, CD11b, CD11c, CD18, CD50, CD54) (20). Indeed, activated γδT cells are efficient antigen-processing and peptide–MHC-presenting cells shown to trigger primary (naïve) and memory responses in both CD4^+^ and CD8^+^ αβT cells (20). Activated γδT cells even trigger antibody production in B cells that may be facilitated by additional co-stimulatory molecules (ICOS, OX40, CD70) (16, 21). Relevant to tumor cell-killing by cytotoxic T cells, activated γδT cells are also able to trigger CD8^+^ αβT cells via antigen cross-presentation, a process involving the uptake and proteasomal processing of exogenous antigen and peptide loading onto intracellular MHC class I molecules (22, 23). Several laboratories have confirmed and extended these initial observations (24–28), corroborating the DC-like properties of short-term activated γδT cells, abbreviated as γδT-APC.

An obvious advantage over DC-based vaccines is the capacity of peripheral blood γδT cells to selectively expand to large numbers during *in vitro* culture in response to HMBPP/IPP or nBP. In fact, numerous studies have already demonstrated that large numbers (10^9^ cells) of γδT cells can be grown from peripheral blood mononuclear cells (PBMC) of patients with cancer, although the level of expansion was shown to be influenced by disease stage and/or γδT cell frequency (29–40). Here, we report that expanded γδT cells maintain critical features of γδT-APC, including DC-like antigen-presenting and co-stimulatory molecules as well as the ability to induce antigen-specific CD4^+^ and CD8^+^ αβT cell responses. A protocol for the preparation of a γδT-APC-based autologous tumor vaccine is discussed.

## Materials and Methods

### Media, reagents, and antibodies

The tissue culture medium used throughout was RPMI 1640 supplemented with 2 mM l-glutamine, 1% non-essential amino acids, 1% sodium pyruvate, 50 μg/ml penicillin/streptomycin (GIBCO Life Technologies, Carlsbad, CA, USA), and combination of 5% FCS (Hyclone Laboratories, Logan, UT, USA) with 5% human serum (prepared in-house). Human recombinant IL-2 (Proleukin) and IL-15 were purchased from Novartis (Basel, Switzerland) and Miltenyi Biotec (Bergisch Gladbach, Germany), respectively. MicroBeads conjugated to mouse monoclonal anti-PE antibodies (Miltenyi Biotec) were used for magnetic cell separation. Mouse monoclonal antibodies (mAbs) specific for CD3 (UCHT1, SK7XX), CD4 (RPA-T4), CD8 (SK1, HIT8a, RPA-T8), CD11a (HI-111), CD14 (M5E2), CD25 (M-A251), CD27 (M-T271), CD45RA (HI100), CD45RO (UCHL-1), CD54 (HA58), CD56 (B159), CD57 (NK-1), CD62L (DREG-56), CD69 (FN50), HLA-DR (L243), CCR7 (3D12), Vδ2-TCR (B6.1), and IFNγ (β27) were from BD Biosciences (San Jose, CA, USA); mouse mAbs specific for TCR-Vγ9 (Immu360) and CD40 (mAB89) were from Beckman Coulter (Brea, CA, USA); mouse mAbs specific for CD28 (28.2), CD86 (IT2.2), CD127 (A019D5), and HLA-ABC (w6/32) were from Biolegend (San Diego, CA, USA); mouse mAb specific for CD19 (SJ25C1), CD25 (BC96), CD80 (2D10.4), NKG2D/CD314 (1D11), and PE-Cy7 conjugated streptavidin were from eBioscience (San Diego, CA, USA); mouse mAb anti TCR-Vγ9 (7A5) was from Thermo Scientific (Waltham, MA, USA); mouse IgG1 mAb (NCG01) was from Abcam (Cambridge, UK); mouse mAb specific for HLA-A2 (BB7.2) was from Serotec (Kidlington, UK). In addition, rat mAb specific for CCR7 (3D12) was from M. Lipp (Max Delbruck Center for Molecular Medicine, Berlin, Germany), and mouse mAb anti-CD18 (TS1-18) was from R. Pardi (Institute San Raffaele, Milano, Italy), and were used together with appropriate isotype controls during flow cytometric analyses. Biotin-SP-conjugated donkey anti-rat IgG was from Jackson ImmunoResearch Laboratories (West Grove, PA, USA). Carboxyfluorescein diacetate succinimidyl ester (CFSE), LIVE/DEAD^®^ Fixable Aqua Dead Cell Stain Kit and Accucheck counting beads were from Invitrogen-Life Technologies (Carlsbad, CA, USA). CellVue and PKH26 were from Sigma-Aldrich (St. Louis, MO, USA).

Peptides were purchased from Severn Biotech (Kidderminster, UK). Recombinant influenza PR8 M1 protein was generated in *E. coli*, isolated to >95% following standard chromatographic procedures and stored in aliquots at −80°C.

### Blood donors

Donors comprised healthy donors and patients with advanced melanoma in the UK and patients with diverse advanced cancers in Italy. The study with venous blood from healthy human individuals was approved by the South East Wales Ethics Committee under reference number 08/WSE04/17. Samples from patients with melanoma, along with anonymized demographic and treatment data, were acquired from the Human Biomaterials Resource Centre (HBRC), University of Birmingham. The HBRC is a biorepository licensed by the Human Tissue Authority, UK and samples were taken with prospective written generic consent for research use. Samples of hematologic malignancies and metastatic colon cancer were obtained from patients enrolled in trials of translational and experimental medicine at Humanitas Research Hospital, Milan, Italy. These protocols have been approved by the Institutional Review Board (IRB) of the same institute (Approvals 1009 for hematologic malignancies and 1021 for metastatic colon cancer), and each patient had signed a consent form that has been approved by the IRB.

### *In vitro* expansion of γδT cells

Whole blood samples from healthy donors and patients with cancer were taken into heparin/EDTA. PBMCs were isolated by Ficoll (Lymphoprep, Axis-Shield, Dundee, UK) gradient centrifugation from whole blood and frozen in liquid nitrogen until use. In order to deplete Treg cells, CD25^+^ cells in PBMC were stained with PE-conjugated anti-CD25 mAb in combination with anti-PE microbeads and then retained on magnetic columns according to the manufacturer’s instructions (Miltenyi Biotec). The pass-through was evaluated for CD25^+^ cell depletion by flow cytometry and designated as CD25^+^ cell depleted PBMC preparations. For *in vitro* γδT cell expansion, PBMC or CD25^+^ cell depleted PBMC were stimulated with 1 μM zoledronate (Zometa; Novartis, Basel, Switzerland) complete RPMI 1640 medium at a density of 10^6^ cells/ml in 24-well plates and cultured at 37°C in 5% CO_2_. At day 5, 8, and 11, cell cultures were split in fresh medium and supplemented with 100 IU/ml IL-2 alone or with 100 IU/ml IL-2 and 20 ng/ml IL-15. At day 14, γδT cell cultures were examined by flow cytometry for γδT cell expansion, purity, and viability as well as expression of cell surface markers.

### Cytokine production

Day 14 expanded γδT cells from PBMC of healthy individuals and melanoma patients were stimulated with 10 nM HMBPP at 1 × 10^6^ cells/well in complete RPMI 1640 medium and cultured for 48 h. Cytokines and chemokines in cell-free culture medium were determined using ELISA kits for IL-1β, IL-8, CCL-1, CCL-4, CXCL13 (R&D Systems, Minneapolis, MN, USA) and IFNγ, TNFα, IL-10, IL-17A, TGFβ1, CCL-2 (eBioscience) and a Dynex MRX II reader (DYNEX Technologies, Chantilly, VA, USA).

### Cytotoxicity assay

Day 14 γδT cell tumor cell-killing activity was tested on the chronic myeloid leukemia cell line KBM7 that was maintained in (1×) Iscove’s Modified Dulbecco’s Medium (GIBCO Life Technologies, Carlsbad, CA, USA) supplemented with 10% fetal calf serum and penicillin/streptomycin. KBM7 cells were labeled with the membrane dye CellVue prior to co-culture with γδT cells. Alternatively, in order to determine the effect of sensitization of KBM7 cells with HMBPP on tumor cell-killing, KBM7 cells were cultured for 18 h in the presence of 1 μM HMBPP and then labeled with the alternative cell membrane dye PKH26 and mixed with untreated CellVue labeled KBM7 cells at a 1:1 ratio. CellVue labeled KBM7 cells or a mixture of Pkh26 labeled, HMBPP-treated KBM7 cells and untreated CellVue labeled KBM7 cells were then incubated together with day 14 γδT cells at increasing effector/target cell ratios for 6 h at 37°C, 5% CO_2_. KBM7 cell-killing was determined by flow cytometry by gating on CellVue^+^ and Aqua^+^ cells or PKH26^+^ and Aqua^+^ cells; Accucheck counting beads were also used for dead cell count.

In order to examine the involvement of γδT cell receptors in KBM7 cell-killing, day 14 γδT cells pre-treated with antibodies to Vγ9-TCR (10 μg/ml), to Vδ2-TCR (1:100 and 1:200 dilution), to CD18 (10 μg/ml), and to NKG2D (10 μg/ml) or control mAb (10 μg/ml) before co-culture with KBM7 cells.

### Antigen-presentation assay

#### Intracellular IFNγ assay

Day 14 γδT cells from HLA-A2^+^ blood donors were incubated for 16–24 h in the presence of 0.01–1 μM influenza M1 protein and then washed and incubated with brefeldin-A pre-treated, HLA-A2^+^ restricted, M1 p58–66-specific CD8^+^ αβT (CD8^+^ responder) cells in 96-well round bottom plates at a ratio of 1:1 (1–0.5 × 10^6^ cells/well) as described (23). Alternatively, day 14 γδT cells were pulsed with 0.1 μM M1 p58–66 and then rigorously washed before use as APC. After 5–6 h, cells were stained with antibodies to CD3, Vγ9-TCR, and CD8, fixed, and then permeabilized in order to detect intracellular IFNγ by flow cytometry. Brefeldin-A treated CD8^+^ responder cells cultured alone or in the presence of PMA and ionomycin served as negative and positive control, respectively (22, 23).

#### Proliferation assays

Day 14 γδT cells from HLA-A2^+^ blood donors were treated with influenza M1 protein or pulsed with M1 p58–66 exactly as described in the intracellular IFNγ assay. Proliferation was measured in M1 p58–66-specific CD8^+^ αβT cells present in freshly prepared, autologous PBMC. Instead of preparing CD3^+^ or CD8^+^ αβT cells for use as responder cells (22, 23), PBMC were directly labeled with 1 μM CFSE for 5 min at room temperature and then washed before use as responder cells. Influenza M1 antigen-treated γδT cells and CFSE-labeled PBMC were mixed at a ratio of 1:5 in 96-well round bottom plates. After 6, 8, and 10 days of culture at 37°C, 5% CO_2_, responder cells were identified by staining with antibodies to CD3, CD8 in combination with M1 p58–66-tetramers; CFSE^low^, M1 p58–66-tetramer^+^ cells were enumerated by flow cytometry. CFSE-labeled PBMC cultured alone or in the presence of day 14 γδT cells that were not pre-treated with influenza M1 antigens served as negative controls. In a second proliferation assay, day 14 γδT cells were treated with 0.01–10 μg/ml *Mycobacterium tuberculosis* purified protein derivative (PPD) before co-culture with autologous, CFSE-labeled PBMC as described for influenza M1 protein. Proliferating (CFSE^low^) cells were detected after day 6, 8, and 10 of culture by staining for activated (CD25^+^) CD3^+^ T cells in combination with either CD4 or CD8 and enumerated by flow cytometry.

### Statistical analysis

Statistical analysis was performed using Student’s *t*-test and results were considered significant at *P*-values of <0.05.

## Results

### Optimized γδT cell expansion during *in vitro* culture

Previous γδT cell expansion studies commonly used IL-2 as T cell growth factor in combination with phosphoantigen or nBP as Vγ9Vδ2-TCR^+^ γδT cell-specific stimuli (29–40). In over 10 individual experiments, we have noticed substantial variations in the yield of γδT cells following the stimulation of PBMC from healthy individuals with zoledronate and their subsequent culture for 14 days in the presence of IL-2. The ability of γδT cells to expand to large numbers is an important aspect in considering their use in immunotherapy and represents a clear advantage over moDC that fail to grow during tissue culture. Therefore, we sought to optimize the culture conditions for reliable expansion of γδT cells by culturing zoledronate-stimulated PBMC from both healthy individuals and patients with cancer in the presence of IL-2 plus IL-15. In addition, due to their inhibitory properties, we examined the effect of removing Treg cells from PBMC on γδT cell proliferation during subsequent tissue culture. Accordingly, cultures were initiated by addition of zoledronate to PBMC either with or without prior depletion of CD25^+^ cells. After 5 days, IL-2 alone or in combination with IL-15 was added and cultures were maintained until day 14 when cells were harvested and analyzed for cell survival, γδT cell content, and purity. As summarized in Table 1, we demonstrate that the combination of IL-2 and IL-15 is approximately 10-fold better than IL-2 alone, yielding >20 × 10^6^ γδT cells per 10^6^ input PBMC in 8 out of 13 γδT cell cultures reaching >70% purity. Of note, consistently higher cell viability was obtained in combination with IL-15 compared to IL-2 alone. Depletion of Treg cells (or CD4^+^ and CD8^+^ T cell subsets; not shown) in input PBMC preparations did not significantly improve the outcome of the IL-2 or IL-2/IL-15 cultures, both in terms of γδT cell purity and cell viability. In our hands, addition of IL-2 right from the start frequently led to substantial αβT cell growth and reduced γδT cell counts in day 14 cultures, which was prevented by delayed (day 5) IL-2 addition (alone or in combination with IL-15).

**Table 1:** ***In vitro* expansion of Vγ9Vδ2-TCR^+^ γδT cells from PBMC of healthy donors in response to zoledronate**.

Treatment	PBMC		CD25^+^ depleted	
	IL-2	IL-2/IL-15	IL-2	IL-2/IL-15
Donors	6	13	6	12
Input of Vγ9^+^ T cells (×10^6^/10^6^ PBMC)^a^	0.019 ± 0.010	0.021 ± 0.011	0.019 ± 0.010	0.022 ± 0.012
**DAY 14 CULTURES**				
Live cells (%)^b^	71 ± 11	85 ± 11	77 ± 9	87 ± 6
CD3^+^ cells (%)^c^	92 ± 10	96 ± 3	94 ± 4	95 ± 4
Vγ9^+^ T cells (%)^c^	56 ± 27	77 ± 21	62 ± 22	74 ± 18
Yield of Vγ9^+^ T cells (×10^6^/10^6^ PBMC)^d^	2 ± 1.2	29 ± 10.4	2.9 ± 0.94	23 ± 8.7
Expansion fold^e^	104 ± 120	1370 ± 949	150 ± 94	1025 ± 721

*^a^ Initiation of cultures at 10^ 6^ PBMC per well (24-well plate)*.

*^b^ Live cells defined as Aqua^−^ cells*.

*^c^ Percentage of total live (Aqua^−^) cells*.

*^d^ Number of Vγ9^+^ T cells per 10^6^ of input PBMC after 14 days of culture*.

*^e^ Yield of Vγ9^+^ T cells divided by input Vγ9^+^ T cells*.

**Figure 1:**
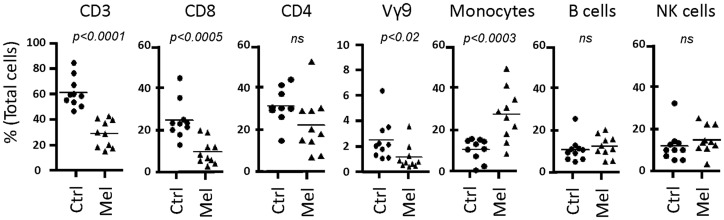
**Cellular composition of PBMC from healthy individuals and melanoma patients**. PBMC were analyzed by flow cytometry for the content of T cells (CD3), T cell subsets (CD8, CD4, Vγ9^+^ γδT cells (Vγ9)), CD14^+^ monocytes, CD19^+^ B cells, and CD3^−^CD56^+^ NK cells, expressed as percent (%) of total live cells in PBMC. Data include PBMC from 10 healthy individuals (Ctrl) and 9 melanoma patients (Mel).

**Figure 2:**
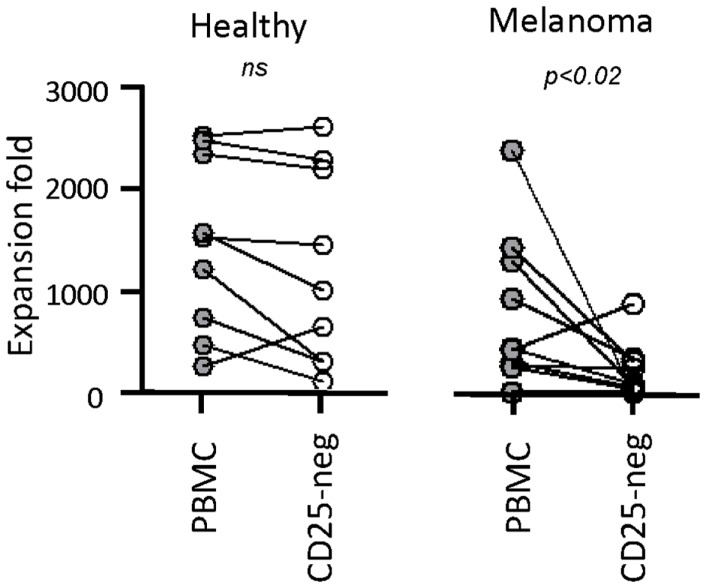
**Negaitve effect of CD25^+^ cell depletion on expansion of γδT cells from melanoma patients**. PBMC from healthy individuals (*n* = 10) and melanoma patients (*n* = 9) were depleted (CD25-neg) or not (PBMC) of Treg cells prior to stimulation with zoledronate plus IL-2/IL-15. Expansion fold is the ratio between the number of γδT cells in day 14 γδT cultures and the input γδT cells, i.e., the number of γδT cells present in PBMC at the beginning of cell culture.

Next, we tested our γδT cell expansion protocol on PBMC from patients with cancer (Table 2). Out of 10 PBMC samples from melanoma patients with advanced disease, one sample with low γδT cell count (0.06% of PBMC) failed to respond during *in vitro* culture. However, the PBMC samples from the remaining nine patients responded well to zoledronate plus IL-2/IL-15 with a range of expansion factors that did not differ substantially from PBMC of healthy donors (Table 3). This finding was somewhat surprising considering the substantial variation in the cellular composition of PBMC from melanoma patients as compared to healthy individuals (Figure 1). For instance, the fraction of CD3^+^ T cells, and most notably CD8^+^ T cells, was consistently lower in PBMC from melanoma patients, whereas the opposite was found for CD14^+^ monocytes. Similarly, the Vγ9^+^ γδT cell count in PBMC from melanoma patients was approximately twofold lower than in PBMC from healthy individuals, which may have been related to the age gap between healthy and melanoma blood donors (41). Reduced γδT cell numbers in the original PBMC samples may have accounted for the twofold lower yields of γδT cells at day 14 of culture (Tables 1 and 3). Of interest, and in clear contrast to a recent report (35), initial depletion of Treg cells in PBMC did not further improve the outcome of γδT cell cultures; quite the contrary, the expansion fold and total yield of γδT cells was substantially and consistently reduced following removal of CD25^+^ cells, which was not observed in control experiments with PBMC from healthy individuals (Figure 2). We have not further examined this observation but suspect that removal of CD25^+^ cells may have led to a reduction in zoledronate-reactive cells in PBMC samples of patients with cancer. Finally, we did not notice a significant difference in γδT cell yield and purity when zoledronate was substituted with IPP or HMBPP (not shown). Overall, our findings document that γδT cells in PBMC from melanoma patients responded well to zoledronate plus IL-2/IL-15. The observed donor-to-donor variations fit well with a recent study carried out with PBMC from 30 melanoma patients, although in the latter study PBMC cultures were terminated at day 7 (42).

**Table 2:** **Melanoma patients’ characteristics**.

Patient	Age (years)	Gender	Date of diagnosis	Disease	Surgery and radiotherapy	Therapy
1	68	F	2008	Lymph nodes and lung metastases	Regional nodal dissection	None
2	65	M	2011	Lung and brain metastases	Regional nodal dissection	None
3	49	M	2012	No current known metastases	Regional nodal dissection; excision distant cutaneous and brain metastases; whole brain radiotherapy	None
4	74	F	2010	Nodal, lung, and brain metastases	Regional nodal dissection	None
5	60	F	2011	Nodal and liver metastases	Regional nodal dissection	None
6	52	F	2011	Distant skin metastases	Regional nodal dissection	None
7	31	F	2007	Skin and lung metastases	Regional nodal dissection	None
8	33	F	2001	Lung metastases	Regional nodal dissection; excision gastro-intestinal metastases	None
9	33	M	2010	Skin, lung, and bone metastases	Regional nodal dissection	Dexame-thasone
10	83	M	2012	Nodal, lung, and liver metastases	Regional nodal dissection	Imatinib

*F, female; M, male*.

**Table 3:** ***In vitro* expansion of Vγ9Vδ2-TCR^+^ T cells from PBMC of melanoma patients in response to zoledronate**.

Treatment of PBMC	IL-2/IL-15	CD25^+^ depleted IL-2/IL-15
Donors	9	9
Input of Vγ9^+^ T cells (x10^6^/10^6^ PBMC)^a^	0.0095 ± 0.01	0.0095 ± 0.01
**DAY 14 CULTURES**		
Live cells (%)^b^	85 ± 7	84 ± 6
CD3^+^ cells (%)^c^	95 ± 4	86 ± 15
Vγ9^+^ T cells (%)^c^	54 ± 33	56 ± 26
Yield of Vγ9^+^ T cells (x10^6^/10^6^ PBMC)^d^	8.2 ± 7.23	2.2 ± 2.72
Expansion fold^e^	861 ± 723	230 ± 272

*^a^ Initiation of cultures at 10^6^ PBMC per well (24-well plate)*.

*^b^ Live cells defined as Aqua^−^ cells*.

*^c^ Percentage of total live (Aqua^−^) cells*.

*^d^ Number of Vγ9^+^ T cells per 10^6^ of input PBMC after 14 days of culture*.

*^e^ Yield of Vγ9^+^ T cells divided by input Vγ9^+^ T cells*.

**Figure 3:**
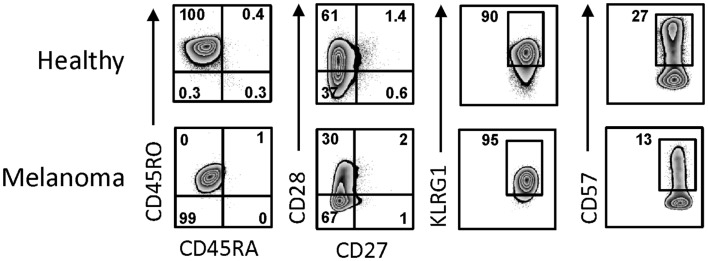
**Activation and differentiation marker expression on day 14 γδT cells from melanoma patients and healthy individuals**. Day 14 γδT cells from PBMC of healthy individuals and melanoma patients were analyzed by flow cytometry for the expression of activation and differentiation markers by gating on Vγ9^+^ cells in combination with the cell surface markers as indicated. One representative example for each of day 14 γδT cell cultures of healthy individuals (*n* = 5) and melanoma patients (*n* = 4) are shown.

Day 14 γδT cells from melanoma patients were also similar to those derived from PBMC of healthy individuals in terms of their activation and differentiation markers (Figure 3). The degree of differentiation was determined by correlating cell surface expression of CD27, CD28, CD45RA, and CD45RO, revealing that all day 14 γδT cells expanded in IL-2/IL-15 expressed the memory marker CD45RO. By contrast, expression of CD27 and CD28 was highly variable in both day 14 γδT cells derived from melanoma patients (3–19 and 2–76%, respectively; *n* = 4) and healthy donors (2–19 and 15–86%, respectively; *n* = 5), reminiscent of effector memory (T_EM_) cells rather than terminally differentiated effector memory (T_EMRA_) cells. CD127, a marker for homeostatic proliferation of αβT cells, was not detected. By contrast, killer cell lectin-like receptor G1 (KLRG1) was present on most expanded γδT cells (Figure 3). The inhibitory receptors PD-1 and CTLA-4 were not detected on day 14 γδT cells of patients or healthy blood donors, whereas the exhaustion marker CD57 was present at various levels (7–64%; *n* = 8). Finally, the combination of homing molecules suggests a preference for inflammatory tissues (CXCR3, CCR4, CCR5, CD11a, CD54, etc.) as opposed to secondary lymphoid organs (CXCR5, CCR7, CD62L) (not shown and see below). We conclude that the addition of IL-2 plus IL-15 to zoledronate-stimulated PBMC resulted in large numbers of *in vitro* expanded γδT cells with a T_EM_ cell-like phenotype while lacking markers commonly associated with inhibitory T cells.

### Responsiveness of expanded γδT cells to phosphoantigens

The use of expanded γδT cells in immunotherapy requires a detailed knowledge about their functionality pertaining to both APC-related and -unrelated cell responses. In agreement with phenotypic marker expression (see above), day 14 γδT cells retained responsiveness to HMBPP as evidenced by cytokine production (Table 4), most notably the pro-inflammatory cytokines IFNγ and TNFα. Secretion of TGFβ and IL-10 was relatively low, yet highly variable and not affected by HMBPP treatment whereas IL-1β, IL-12, and IL-17 were not detected. In addition, the chemokines CXCL8, CCL1, and CCL4 were found to be present. Again, cytokine secretion profiles did not differ substantially between day 14 γδT cells derived from healthy individuals as compared to melanoma patients. As expected (43), zoledronate alone was inactive and cytokine responses were only seen when zoledronate plus monocytes were added to day 14 γδT cells (not shown).

**Table 4:** **Secretion of cytokines by day 14 γδT cells following stimulation with PA**.

		Cytokines^c^ (pg/ml)			
	Stimulation^b^	IFNγ	TNFα	TGFβ	IL-10
Healthy^a^	–	7900 ± 7600	23 ± 24	77 ± 97	129 ± 58
	HMBPP	32000 ± 22000	550 ± 427	32 ± 55	95 ± 57
Melanoma^a^	–	2800 ± 1700	20 ± 6	238 ± 172	183 ± 317^d^
	HMBPP	26000 ± 7000	130 ± 64	247 ± 45	205 ± 353^d^

*^a^ Day 14 γδT cells from PBMC of either three healthy individuals or three melanoma patients*.

*^b^ Treatment of day 14 γδT cells with or without 10 nM HMBPP*.

*^c^ Cytokines determined by ELISA*.

*^d^ Two out of three melanoma γδT cell cultures has no (0 pg/ml) IL-10, irrespective of HMBPP treatment*.

Functionality of day 14 γδT cells was also evidenced in their proliferation responses. In the presence of IL-2 and IL-15 but in the absence of HMBPP, day 14 γδT cells continued to proliferate at variable levels (zero to sixfold) during subsequent 7 days cultures, which may reflect the considerable variation in activation states, as evidenced by the expression of CD69 [75–86 and 24–96% in γδT cells from healthy individuals (*n* = 5) and melanoma patients (*n* = 9), respectively] and CD25 [2–36 and 2–76% in γδT cells from healthy individuals (*n* = 5) and melanoma patients (*n* = 9), respectively]. Addition of HMBPP killed >50% of day 14 γδT cells within 24 h, but the residual cells resumed proliferation to levels routinely exceeding the ones seen in cultures without addition of HMBPP.

**Figure 4:**
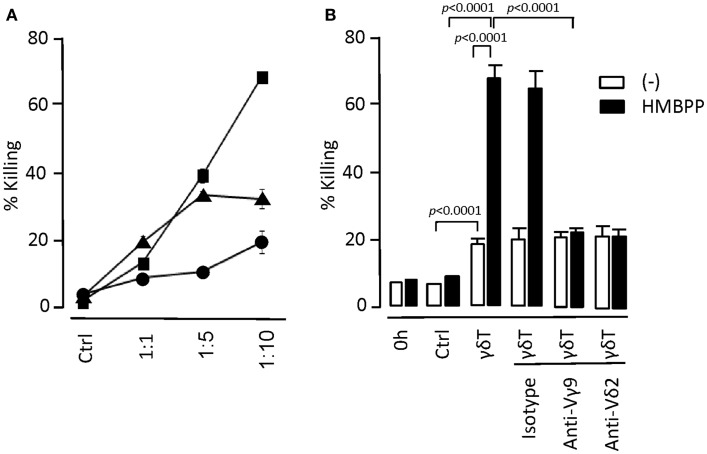
***In vitro* tumor cell-killing by day 14 γδT cells**. **(A)** Myeloid leukemia KBM7 target cells were labeled with the cell membrane dye PKH26 and mixed with day 14 γδT cells at target to effector cell ratios as indicated. After 6 h of incubation at 37°C, remaining live target cells were determined by including flow cytometry counting beads. Percent (%) killing refers to the ratio of remaining to input target cells × 100. As control (Ctrl), targets cells were cultured for 6 h in the absence of γδT cells; representative of three experiments. **(B)** KBM7 target cells were cultured overnight in the presence (+HMBPP) or absence (−) of 1 μM HMBPP. The two cultures were labeled with either PKH26 or Cellvue and added to day 14 γδT cells at a target to effector cell ratio of 1:10. To examine the mechanisms of target cell-killing, antibodies to γδTCR (anti-Vγ9; 10 μg/ml and anti-Vδ2; 1/100 dilution) and isotype antibody (mouse IgG1; 10 μg/ml) were included in the cultures. After 6 h of culture, percent killing was determined as outlined above by gating on PKH26^+^ (HMBPP-treated KBM7) and CellVue^+^ (control KBM7) cells; representative of two experiments.

We also examined *in vitro* tumor cell-killing activity by day 14 γδT cells. The underlying mechanisms are still debated but have been proposed to involve either γδTCR- or NK receptors-mediated processes or a combination of both pathways. We demonstrate that day 14 expanded γδT cells retained tumor cell-killing activity without the need for prior activation (Figure 4). However, the target tumor cells (myeloid KBM7 cells) were much more efficiently killed following overnight incubation with HMBPP. This effect was reduced to the level of untreated KBM7 cells when γδTCR-blocking Abs were included, demonstrating that the HMBPP pretreatment of tumor cells directly promoted the γδTCR-mediated KBM7 killing. γδTCR-blocking Abs did not affect the killing of untreated KBM7 cells whereas the addition of CD18- or NKG2D-blocking Abs reduced the killing of both untreated and HMBPP-pre-treated KBM7 cells (not shown). We conclude that day 14 γδT cells retained their ability to respond to Vγ9Vδ2-TCR-specific ligands and were able to generate cell responses that were typically seen with freshly isolated γδT cells.

### Antigen-presentation by expanded γδT cells (γδT-APCs)

**Figure 5:**
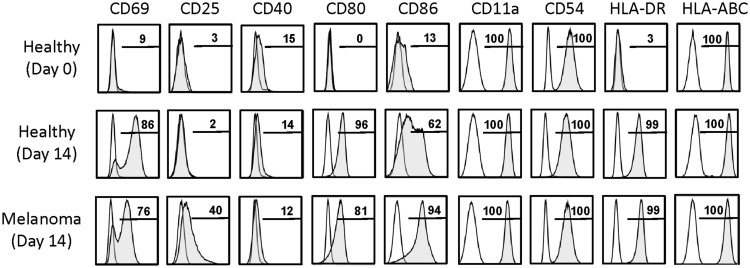
**Activation and APC marker expression on day 14 γδT cells**. Cell surface markers as indicated were determined by flow cytometry on (Vγ9^+^) γδT cells present in freshly isolated PBMC of healthy individuals (Healthy, day 0) and in day 14 γδT cell cultures derived from PBMC of healthy individuals (Healthy, day 14) or melanoma patients (Melanoma, day 14). Representative data of 6 (Healthy, day 0), 5 (Healthy, day 14), and 4 (Melanoma, day 14) experiments are shown.

The purpose of this study was to determine whether expanded γδT cells could be potentially used as a novel type of cellular vaccine for the treatment of patients with cancer. Therefore, we sought to establish whether day 14 γδT cells, as opposed to short-term activated γδT cells (20, 22, 23), expressed APC markers and were able to induce *in vitro* antigen-specific αβT cell responses. A hallmark of γδT-APC generated during short-term activation of peripheral blood γδT cells was their high-level expression of antigen-presentation (MHC I and II), co-stimulation (CD40, CD80, CD86, etc.), adhesion (CD11a, CD54, etc.), and lymph node homing (CD62L, CCR7) molecules (20). Of interest, and in clear contrast to γδT cells freshly isolated from peripheral blood, day 14 γδT cells retained many of these APC markers, most notably CD80, CD86, and HLA-DR (MHC II) (Figure 5). The adhesion molecules CD11a and CD54 were still uniformly expressed whereas CD40 and CCR7 (not shown) were low or absent. Maintenance of crucial APC markers may well reflect the presence (albeit at various levels) of activation (CD69) and/or proliferation (CD25) markers, which were low/absent on resting (day 0) γδT cells. Of interest, re-stimulation of day 14 γδT cells with HMBPP for 3 days resulted in moderate CCR7 and CD62L expression (4–16% of cells), indicating that extensively expanded γδT cells retained in part the ability to home to secondary lymphoid tissues. Together, these data demonstrate that day 14 γδT cells retained many APC markers that are routinely induced in short-term activated γδT cells and, importantly, that APC marker expression did not differ between day 14 γδT cells derived from healthy individuals and melanoma patients. In fact, preliminary data with PBMC from melanoma-unrelated patients with cancer, including patients with Hodgkin’s lymphoma (HL), non-Hodgkin’s lymphoma (NHL), and colorectal carcinoma (CRC), revealed similar APC marker expression on expanded γδT cells (not shown), indicating that the APC phenotype is a general characteristic of expanded γδT cells.

**Figure 6:**
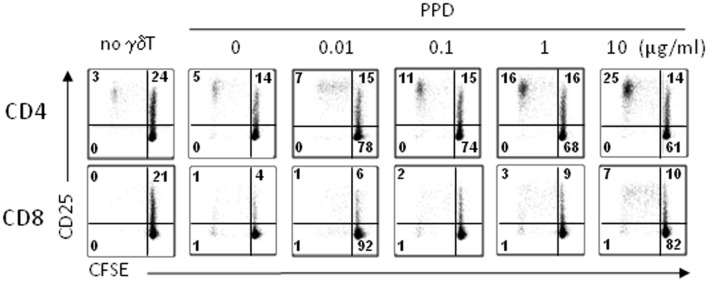
**Proliferation responses to PPD-treated day 14 γδT cells**. Day 14 γδT cells from healthy individuals were treated overnight with PPD at concentrations as indicated, washed, and mixed with freshly isolated, CFSE-labeled PBMC from donor-matched blood samples at a γδT cell to PBMC ratio of 1:5. At day 6 (and days 8 and 10; not shown), proliferating CD4^+^ and CD8^+^ αβT cells were identified by flow cytometry in the Vγ9^−^ CD3^+^ T cell gate as activated (CD25^+^) CFSE^low^ cells. Control cultures (no γδT) contained CFSE-labeled PBMC but not γδT cells. One representative of four independent experiments is shown.

Antigen-presenting cell marker expression suggested that day 14 γδT cells would function as APC without the need for re-stimulation, i.e., treatment with phosphoantigens, which is in clear contrast to γδT cells freshly isolated from peripheral blood that required stimulation (20). First, we tested the ability of day 14 γδT cells to induce αβT cell proliferation in response to *M. tuberculosis* PPD as carried out previously (20). To mimic the situation of a first-in-man clinical trial, we used unfractionated PBMC (as opposed to purified αβT cells) as responder cells. Following treatment with PPD, day 14 γδT cells were mixed with 5-(and 6-)carboxyfluorescein diacetate succinimidyl ester (CFSE)-labeled, autologous PBMC and proliferating CD4^+^ and CD8^+^ αβT cells were identified as CD25^+^ CFSE^low^ cells at d4, d6, and d8 of culture. The representative example in Figure 6 clearly demonstrates that day 14 γδT cells were able to process PPD and present PPD-derived peptides on MHC class I and II to CD8^+^ and CD4^+^ αβT cells, respectively, at PPD concentrations as low as 0.1 μg/ml. Furthermore, CD4^+^ αβT cells proliferated much better than CD8^+^ αβT cells as evidenced by their higher background (absence of γδT cells) and maximal responses (γδT cells treated with 10 μg/ml PPD). Although clearly evident, CD8^+^ αβT cell responses were smaller, which may reflect the inefficiency of antigen cross-presentation, a lower frequency of PPD-specific CD8^+^ memory T cells among PBMC, lack of endogenous IL-2 production required for proliferation or a combination of these factors.

**Figure 7:**
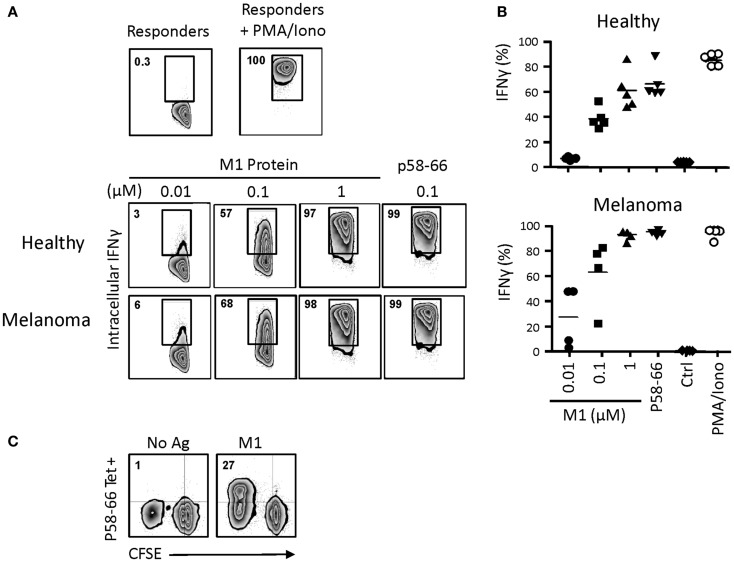
**Influenza M1 cross-presentation to CD8+ αβT cells by day 14 γδT cells**. **(A)** Day 14 γδT cells from HLA-A2+ healthy individuals or melanoma patients were treated overnight with influenza M1 protein at concentrations as indicated, washed, and mixed in equal numbers with a HLA-A2-restricted, p58–66-specific CD8+ αβT cell line. In control experiments, day 14 γδT cells were directly pulsed with 0.1 μM M1 peptide p58–66, washed, and then used in the APC assay. After 6 h of incubation, intracellular IFNγ production in M1 p58–66-tetramer + responder cells was determined by flow cytometry. Responder cells cultured in the absence of stimulation or in the presence of PMA and ionomycin served as controls for background and maximal IFNγ responses. **(B)** Compilation of intracellular IFNγ responses obtained with day 14 γδT cells from PBMC of five HLA-A2+ healthy individuals and four HLA-A2+ melanoma patients. **(C)** Day 14 γδT cells from HLA-A2+ healthy individuals were treated with 1 μM M1 protein (M1) or not (No Ag) as described in **(A)** and then mixed with donor matched, CFSE-labeled PBMC at a γδT cell to PBMC ratio of 1:5. Proliferation responses in p58–66-tereamer + CD8 + αβT cells were determined essentially as described in Figure 6. One representative of two independent experiments is shown.

In order to examine antigen cross-presentation in more detail, we incubated day 14 γδT cells with the influenza M1 protein, which requires proteasomal processing and loading of the M1 immunodominant peptide p58–66 onto intracellular HMC class I (HLA-A2) molecules (22, 23). Following 24 h incubation with graded concentrations of M1 protein, day 14 γδT cells were washed and incubated with M1 responder T cells (HLA-A2^+^, M1p58-66-specific CD8^+^ αβT cells). After 6 h, intracellular IFNγ production in responder cells was measured by flow cytometry (Figure 7A). Processing of 1 and 0.1 μM M1 protein by day 14 γδT cells led to robust IFNγ responses, similar to what we have published with short-term activated γδT cells (22, 23). The nine amino acid M1p58–66 peptide can be pulsed directly (without the need of prior uptake and intracellular processing) onto cell surface HLA-A2 on APCs such as DC or, as shown here, γδT cells. Thus, M1p58–66-pulsed, day 14 HLA-A2^+^ γδT cells initiated maximal intracellular IFNγ responses. Day 14 γδT cells from HLA-A2-individuals were unable to present the M1p58–66 peptide and, thus, served as negative control (not shown). Of note, we did not detect substantial differences with day 14 γδT cells derived from either healthy individuals or melanoma patients in their potencies to induce intracellular IFNγ responses (Figure 7B). Also, donor-matched monocyte-derive DC from PBMC of healthy individuals gave similar results, although these experiments were carried out on separate days (7 days after PBMC isolation; data not shown). The influenza results were replicated with CMV UL83 peptides and UL83 p495–503-specific CD8^+^ αβT responder cells (not shown). Moreover, day 14 γδT cells from melanoma-unrelated patients (HL, NHL and CRC; see above) were also capable of cross-presenting influenza M1 p58–66 to CD8^+^ αβT responder cells (not shown). Finally, we investigated the ability of day 14 γδT cells to induce influenza-specific proliferation responses in primary CD8^+^ αβT cells (as opposed to cultured cell lines) in unfractionated PBMC as described above. Data in Figure 7C show that influenza M1 treated, day 14 γδT cells but not untreated day 14 γδT cells induced robust proliferation responses in p58–66-specific CD8^+^ αβT cells. In fact, half of all proliferating (CFSE^low^) cells were p58–66-specific whereas these cells did not expand at all in response to untreated day 14 γδT cells, underscoring the M1p58–66 specificity of this response. We conclude that day 14 γδT cells behaved similarly to short-term activated γδT cells (20) in their ability to process simple (influenza M1) and complex (PPD) antigens and to induce antigen-specific CD8^+^ and CD4^+^ αβT cell responses. Equally important, day 14 γδT cells from patients with cancer maintained their APC functionality, suggesting that patients-derived γδT cells may provide a valuable source for the generation of an autologous cellular vaccine.

## Discussion

The principal aim of the present study was the detailed characterization of the APC properties of γδT cells following their expansion during *in vitro* culture. It is already well known that Vγ9Vδ2-TCR^+^ γδT cells in PBMC respond vigorously to phosphoantigens or nBP leading to extremely large numbers during *in vitro* culture in the presence of T cell growth factors (29–40). Of note, treatment of PBMC with phosphoantigen or nBP is highly selective for blood γδT cells such that unrelated lymphocytes, including αβT cells and NK cells, do not respond and are “left behind” during *in vitro* culture. This simple technique represents a considerable advantage over αβT cells when large numbers are needed for autologous cell therapy. Since γδT cells themselves do not produce sufficient IL-2, this or other T cell growth factors need to be included during *in vitro* γδT cell expansion. In our hands, delayed addition of IL-2 prevented early expansion of unrelated lymphocytes and, consequently, prevented outgrowth of αβT cells or NK cells. Since our previous work with IL-2 provided inconsistent results in terms of yield and purity of expanded γδT cells, we decided to modify the *in vitro* culture procedure by including either IL-15 or by depleting Treg cells prior to cell culture. In short, Treg cell depletion, i.e., removal of CD25^+^ cells by MACS beads isolation columns, did not affect γδT cell cultures from PBMC of healthy individuals whereas the addition of IL-15 was clearly beneficial (see below). Interestingly, we observed that removal of CD25^+^ cells from PBMC of melanoma patients was inhibitory, supporting the view that this treatment removed zoledronate-responsive cells that were required for optimal γδT cell activation. In fact, PBMC from patients with cancer contained numerous CD25^+^ monocytes, which were rare in PBMC from healthy individuals, suggesting that their removal might have contributed to the reduced responsiveness to zoledronate.

IL-2 is known to induce terminal T cell differentiation and, eventually, activation-induced cell death, which may limit the expansion and/or viability of cultured T cells (44). By contrast, and in addition to its T cell growth factor properties, IL-15 induces T cell survival (45, 46) and, in CD8^+^ αβT cells, enhances cytotoxic functions (47, 48). IL-15 alone or in combination with IL-2 has been used successfully in cultures of tumor-infiltrating αβT cells isolated from melanoma (49) and Merkel cell carcinoma patients (50). The action of IL-15 on human blood γδT cells is not well understood. In agreement with its involvement in homeostatic proliferation of γδT cells in mice (51, 52), persistence of γδT cells in patients with cancer has been explained by the presence of endogenous IL-15 (53). Here, we report that the combination of IL-2 and IL-15 made a substantial difference as compared to IL-2 alone during culturing of γδT cells derived from both patients and healthy blood donors.

A high yield of expanded, autologous γδT cells is certainly desirable for their further use as cellular vaccine. However, the quality of expanded γδT cells in terms of their state of differentiation and responsiveness to re-stimulation are equally important parameters to consider. Day 14 γδT cells bear surface markers reminiscent of T_EM_ cells, although the validity of adopting this terminology for γδT cells is questionable. T_EM_ cells and central-memory T (T_CM_) cells describe subsets of memory αβT cells present in peripheral blood and were originally defined on the basis of the expression of lymph node-homing markers (CD62L, CCR7) (54). Memory subsets, most notably CD8^+^ αβT cell subsets, were further defined by the expression of CD45RA and CD45RO in combination with co-stimulatory receptors (CD27, CD28) (55). Except for CCR7/CD27/CD28 triple-negative CD8^+^ αβT cells, all T cell subsets purely reflect a hallmark of αβT cell biology, namely their need to interact with DC in lymph nodes or peripheral tissues in order to receive appropriate activation/differentiation signals. γδT cells, including the dominant subset of Vγ9Vδ2-TCR^+^ γδT cells in peripheral blood, are not controlled by DC in secondary lymphoid tissues, suggesting fundamental mechanistic differences in controlling responsiveness between these two classes of T cells. Nevertheless, day 14 γδT cells share hallmarks of late-differentiated T_EM_ cells with intact replication capacity because they lack CD45RA, CCR7, and for the most part CD27 but largely maintain CD28 expression. Uniform expression of KLRG1, otherwise a marker for replication-defective CD8^+^ αβT cells (56), may signify a role for these cells in epithelial tissues where the KLRG1 ligand, E-cadherin, is ubiquitously expressed (57). In agreement, day 14 γδT cells responded well to re-stimulation with HMBPP or zoledronate plus PBMC as assessed by cytokine production and γδT cell proliferation. Of note, day 14 γδT cells primarily produced pro-inflammatory cytokines (IFNγ, TNFα), much like activated peripheral blood γδT cells (58) but failed to secrete regulatory cytokines TGFβ and IL-10 in response to HMBPP or to express the inhibitory receptors PD-1 and CTLA-4. Day 14 γδT cells also maintained tumor cell-killing activity, most notably when HMBPP-pre-treated tumor cells were used as target cells. Collectively, stimulation of PBMC with zoledronate and culturing in the presence of IL-2 plus IL-15 resulted in large numbers of functionally competent γδT cells featuring immunostimulatory and cytotoxic as opposed to inhibitory properties.

Most relevant for the potential use of expanded γδT cell as a novel cellular vaccine were the abundant expression of APC-relevant cell surface markers and the capacity of day 14 γδT cells to trigger antigen-specific αβT cell responses *in vitro*. Professional APC, such as DC, have to be able to initiate adhesive interactions with αβT cells in order to allow αβT cells to screen the APC surface for the presence of their cognate peptide–MHC complexes (59). Engagement of αβTCR on CD8^+^ and CD4^+^ αβT cells with peptide–MHC class I and class II complexes on DC, respectively, leads to αβT cells priming and subsequent engagement of co-stimulatory receptors initiates αβT cell proliferation and differentiation. Interference in any of these early steps has profound effects on αβT cell responses. We here show that day 14 γδT cells abundantly express CD11a, CD54 (adhesion) and HLA-DR and HLA-ABC (antigen-presentation) molecules as well as the CD28 ligands CD80 and CD86, which trigger essential CD28-mediated co-stimulatory signals in αβT cells (60). Indeed, this APC phenotype is mirrored by the APC function, as demonstrated by the induction of antigen-specific CD4^+^ and CD8^+^ αβT cell proliferation in response to expanded γδT cells that have processed either the complex microbial antigen PPD or influenza M1 protein. We did not notice significant differences between day 14 γδT cells derived from PBMC of healthy individuals as compared to patients with cancer with regard to their ability to cross-present M1 protein. Of note, the cellular composition of PBMC from melanoma patients differed substantially from PBMC of healthy individuals, possibly reflecting the history of cancer treatment regimens and potential immunosuppressive conditions (61). However, day 14 γδT cells have undergone many rounds of cell division and, thus, appear “far removed” from the inhibitory conditions present in the original PBMC samples, which may explain their undiminished functionality. In this respect, our data confirm previous findings about γδT cell-induced CD8^+^ αβT cell responses although patients-derived γδT cells were examined at their peak-time (day 7/8) of proliferation (25, 27).

In summary, we demonstrated that expanded γδT cells from PBMC of both healthy individuals and patients with cancer displayed potent APC functions and responsiveness to γδT cell antigens. Our data support the view that expanded γδT cells may be exploited as a novel cellular vaccine for the treatment of patients with cancer. Relatively small (≈100 ml) blood samples of patients suffice to generate large numbers (>10^9^) of expanded γδT cells within 2 weeks of *in vitro* culture in response of zoledronate and the T cell growth and survival factors IL-2 and IL-15. Cultured γδT cells efficiently take up, process, and cross-present complex antigens (20, 22), including proteins present in cell extracts (23). Therefore, we propose to treat expanded γδT cells with tumor antigens, either in the form of cellular extracts derived from patients’ own tumor cells or from heterologous tumor cell lines. The latter approach would also provide highly stimulatory alloantigens that may boost the adaptive immune response. Tumor-antigen-loaded γδT cells are then infused into patients. Since expanded γδT cells retain the capacity to secrete pro-inflammatory cytokines (IFNγ, TNFα) and to express, albeit at reduced levels, lymph node-homing markers (CCR7, CD62L), the infusion of tumor-antigen-loaded γδT cells may be followed by a single treatment with zoledronate. Thus, a fraction of activated γδT cells may acquire the ability to home to secondary lymphoid tissues (spleen, lymph nodes), where mobilization of endogenous tumor-specific αβT cells takes place. The anti-tumor activity mediated by this new wave of endogenous effector T cells may be further supported by the infused γδT cells that retained direct tumor cell-killing activity. A substantial body of evidence by numerous laboratories has already documented the safety of large (and repetitive) infusions of γδT cells (33, 37, 38, 62–67). Now, we need to test whether the clinical outcome is improved by treating patients with tumor-antigen-presenting γδT cells targeting the patients’ own tumor-specific T cells as opposed to tumor cells. It is possible that the immune stimulatory effects of γδT cell-based vaccines are further enhanced when applied in combination with promising immune checkpoint inhibitors (anti-PD-1 and anti-CTLA-4 antibodies) (68).

## Conflict of Interest Statement

The authors declare that the research was conducted in the absence of any commercial or financial relationships that could be construed as a potential conflict of interest.
